# Increased concentrations of platelet- and endothelial-derived microparticles in patients with myocardial infarction and reduced renal function- a descriptive study

**DOI:** 10.1186/s12882-019-1261-x

**Published:** 2019-03-01

**Authors:** Josefin Mörtberg, Kristina Lundwall, Fariborz Mobarrez, Håkan Wallén, Stefan H. Jacobson, Jonas Spaak

**Affiliations:** 1 Division of Nephrology, Department of Clinical Sciences, Danderyd University Hospital, Karolinska Institutet, Stockholm, Sweden; 2 Division of Cardiovascular Medicine, Department of Clinical Sciences, Danderyd University Hospital, Karolinska Institutet, Stockholm, Sweden; 3 Rheumatology Unit, Department of Medicine, Solna, Karolinska Institutet, Karolinska University Hospital, Stockholm, Sweden

**Keywords:** Renal dysfunction, Renal failure, Chronic kidney disease, Acute coronary syndrome, Thrombosis, Haemostasis

## Abstract

**Background:**

Patients with chronic kidney disease (CKD) have a high risk of recurring thrombotic events following acute myocardial infarction (AMI). Microparticles (MPs) are circulating small vesicles shed from various cells. Platelet microparticles (PMPs) reflect platelet activation and endothelial microparticles (EMPs) reflect endothelial activation or dysfunction. Both increase following AMI, and may mediate important biological effects. We hypothesized that AMI patients with CKD have further elevated PMPs and EMPs compared with non-CKD patients, despite concurrent antithrombotic treatment.

**Methods:**

We performed a descriptive study of patients with AMI. Fasting blood samples were acquired from 47 patients on dual antiplatelet treatment. Patients were stratified by renal function: normal (H; *n* = 19) mean eGFR 88; moderate CKD (CKD3; *n* = 15) mean eGFR 47, and severe CKD (CKD4–5; *n* = 13) mean eGFR 20 mL/min/1.73 m^2^.

MPs were measured by flow-cytometry and phenotyped according to size (< 1.0 μm) and expression of CD41 (GPIIb; PMPs) and CD62E (E-selectin; EMPs). In addition, expression of platelet activation markers P-selectin (CD62P) and CD40ligand (CD154) were also investigated.

**Results:**

PMPs expressing CD40 ligand were higher in CKD4–5: 210 /μl (174–237); median and interquartile range; vs. group H; 101 /μl (71–134; *p* < 0.0001) and CKD 3: 142 /μl (125–187; *p* = 0.006). PMPs expressing P-selectin were higher in CKD4–5 compared with H, but not in CKD3. EMPs were higher in CKD4–5; 245 /μl (189–308) compared with H; 83 /μl (53–140; p < 0.0001) and CKD3; 197 /μl (120–245; *p* < 0.002).

**Conclusions:**

In AMI patients, PMPs and EMPs from activated platelets and endothelial cell are further elevated in CKD patients. This indicate impaired endothelial function and higher platelet activation in CKD patients, despite concurrent antiplatelet treatment.

## Background

Chronic kidney disease (CKD) is a large and growing health problem which affects 10–18% of the population in the western world [1, 2]. CKD patients have a markedly increased risk to develop cardiovascular disease (CVD) including acute myocardial infarction (AMI) [3, 4]. They also have an increased risk of mortality and morbidity after a cardiovascular event, such as AMI, stroke, and following coronary artery bypass graft surgery [5–7]. This is only partially explained by a high prevalence of traditional Framingham risk factors such as smoking, dyslipidaemia, hypertension, hyperglycaemia and obesity. In CKD, non-traditional risk factors including oxidative stress, inflammation, malnutrition, anaemia, endothelial dysfunction, hyper-homocysteinemia, and abnormal calcium phosphate metabolism have all been shown to contribute [8, 9]. CKD patients also have a disturbed haemostasis shifted towards a prothrombotic state [10]. At the same time as CKD patients have a high risk of thrombotic events, they also have an increased risk of bleeding [11]. This may in part explain why CKD patients are less likely to receive early revascularisation when they have an AMI, and receive less evidence based secondary prevention treatment [12, 13].

Microparticles (MPs) are small sized vesicles 0.1–1.0 μm circulating in blood under normal physiological conditions. They are shed from various cell types such as platelets, endothelial cells and leucocytes, in particular when cells are activated or during apoptosis. Activation occurs mainly by different chemical stimuli or shear stress. MPs can carry bioactive surface- and intracellular proteins originating from the parent cells.

MPs are described as future biomarkers for various diseases. Recent studies have shown that they also actively induce biological responses and inter-cellular cross-talk [14–16]. MPs may expose phosphatidylserine (PS) on the outer surface, offering a negatively charged surface that promotes coagulation and fibrin formation [17]. They are elevated in several diseases, especially those associated with vascular injury, inflammation and prothrombosis, such as diabetes mellitus, CKD, hypertension, atherosclerosis and following an AMI [18–20].

Platelet derived MPs (PMPs) are the most abundant in the circulation, followed by endothelial derived microparticles (EMPs).

PMPs carry several proteins and cytokines expressed on the surface of platelets, such as integrin glycoprotein (GP) IIb/IIIa (CD41/61), GPIX (CD42a), GPIb (CD42b) as well as platelet activation markers P-selectin (CD62P), and CD40 ligand (CD154). All of which can promote PMPs an ability to recruit inflammatory cells, especially leukocytes and monocytes, induce adhesion to endothelial cells, and influence haemostasis [14, 17]. PMPs appear to have a direct effect on platelet aggregation and haemostasis [21], and have an ability to decrease fibrin network permeability in vitro [22]. Circulating PMP concentrations increase in patients with various diseases such as AMI, diabetes mellitus, hypertension, asymptomatic atherosclerotic lesions, and in CKD [18–20, 23].

Endothelial microparticles (EMPs) are released upon endothelial cell activation or apoptosis, and therefore express activation molecules on their surface, such as E-selectin (CD62E), vascular cell adhesion molecules (VCAM-1) and intercellular adhesion molecules (ICAM-1). Endothelial dysfunction is an early hallmark of atherosclerosis and cardiovascular disease [24]. EMPs correlate with the level of endothelial activation both in CVD and CKD, and a high level of endothelial MP shedding may reflect dysfunction or damage [23, 25, 26]. In line with this there seem to be a correlation between levels of EMPs and risk of cardiovascular events and death [27–30].

Thus, PMPs and EMPs are markers of an activated state, and are shown to be useful biomarkers of atherosclerosis and cardiovascular disease [20]. Growing evidence indicate that their biological effect may contribute to an increased risk of recurring thrombotic events in CKD patients, and might be an important link between endothelial dysfunction and a pro-thrombosis state.

CKD patients have more recurrent thromboembolic events and worse prognosis after an AMI, and current antithrombotic treatments may be less efficacious in these patients. In order to study this, we assessed platelet and endothelial activation by measuring circulating PMPs and EMPs in AMI patients with normal and reduced renal function.

## Methods

### Patients and study design

In this descriptive study we used plasma samples from the Stockholm heart bank biobank (NCT01204645) including patients donating blood during an AMI. The AMI diagnosis was decided by routine evaluation of clinical history, ECG and Trop I levels. Renal function was estimated using the CKD-EPI formula in mL/min/1.73m^2^. We stratified the patients in the biobank (*n* = 811) according to their renal function: non CKD (H), CKD 3 and CKD 4–5, and then selected 20 patients consecutively from each of the groups. After excluding patients on dialysis, transplanted patients, and patients without treatment with antiplatelet therapy P2Y12 receptor antagonists, 47 patients remained. Patients with normal renal function (H; *n* = 19) had a mean eGFR of 88 mL/min/1.73 m2; moderate CKD (CKD 3; *n* = 15) mean eGFR 47 mL/min/1.73 m2, and severe CKD (CKD 4–5; *n* = 13) mean eGFR 20 mL/min/1.73 m2.

All patient’s previous medical records were examined to ensure that the renal function at admission reflected their previous levels, and that they fulfilled the criteria for a chronic kidney disease diagnosis.

The patients were included in the biobank between 2010 and 2015. Analysis were performed in 2016–17. The study was approved by the Ethics committee of the Karolinska Institutet, Stockholm, Sweden. Baseline characteristics are listed in Table 1.

**Table 1:** Patient characteristics

	Normal kidney function (H)	CKD 3	CDK 4–5
Subjects	n = 19	n = 15	n = 13
Mean eGFR (mL/min/1.73m^2^)	88	47	20
Mean Age (years)	66	76	74
Sex ratio Female/Male	31%F (6F/13M)	13%F (2F/13M)	40% (5F/8M)
Smoking	32% (6/19)	20%(3/15)	8% (1/13)
Diabetes mellitus 2	21% (4/19)	40% (6/15)	69% (9/13)
STEMI	47% (9/19)	47% (7/15)	46% (6/13)
Non-STEMI	52% (10/19)	53% (8/15)	54% (7/13)
Angiography before sampling	89% (17/19)	85% (11/15)	77% (10/13)
PCI before sampling	68% (13/19)	40% (6/15)	69% (9/13)
Angiography after sampling	5% (1/19)	20% (3/15)	15% (2/13)
No angiography	5% (1/19)	7% (1/15)	8% (1/13)
PCI anytime	74% (14/19)	47% (7/15)	77% (10/13)
Hypertension	58% (11/19)	93% (14/15)	100% (13/13)
Chronic ischemic heart disease	26% (5/19)	47% (7/15)	69% (9/13)
Sample day	Mean 2.6	Mean 2.6	Mean 3.9
Medications:			
Acetylsalicylic acid	95% (18/19)	93% (14/15)	100% (13/13)
P2Y_12_ receptor antagonists	100%	100%	100% (13/13)
Clopidogrel	84% (16/19)	85% (11/15)	92%(12/13)
Ticagelor	11% (2/19)	20% (3/15)	15%(2/13)
Prasugrel	5% (1/19)	7% (1/15)	0% (0)
LMWH	42% (8/19)	53% (8/15)	23% (3/13)
Warfarin	0	0	8% (1/13)
NOAK	0	0	0
Statin	84% (16/19)	87% (13/15)	85% (11/13)
Laboratory parameters:			
Mean Platelet count 10^9^/L	212 ± 57	188 ± 54	205 ± 83
Mean Leucocyte count 10^9^/L	9,0 ± 2.7	7.6 ± 1.3	11.5 ± 5.7
Mean p-albumin (g/L)	35 ± 3	33 ± 3	30 ± 7
Mean p-phosphate (mmol/L)	1.06 ± 0.16	1.05 ± 0.15	1.22 ± 0.37

### Blood samples

All samples were acquired fasting in the morning 1–3 days after admission for an AMI. Antecubital venous blood was sampled in test tubes containing 1/10 0.129 M sodium citrate. Platelet poor plasma (PPP) was collected after centrifugation at 2000 G for 20 min at room temperature (RT) and then frozen in aliquots at − 80 °C until analysis. Centrifugation started within 20 min after the blood was drawn.

### Microparticle analysis

PPP was later thawed and was initially centrifuged at 2 000 G for 20 min at RT. The supernatant was centrifuged again at 13 000 G for 2 min. Subsequently, 20 μl of the supernatant was incubated for 20 min in dark with lactadherin-FITC (Coatech AB; BLAC-FITC), CD41-APC or PC7 (Beckman Coulter, Brea, CA, USA), CD62P-PE (Thermo Fisher Scientific, Waltham, MA, USA), CD154-APC (Thermo Fisher Scientific, Waltham, MA, USA) and CD62E-APC (Thermo Fisher Scientific, Waltham, MA, USA). MPs were defined as particles less than 1.0 μm in size and positive to lactadherin (binds to exposed phosphatidylserine) and cell-markers described above. All samples were analysed using a Beckman Coulter Gallios flow cytometer (Beckman Coulter, Brea, CA, USA), and the MP-gate was calibrated using Megamix beads (FSC; 0.3 μm, 0.5 μm and 0.9 μm BioCytex, Marseille, FR). In the present study results are shown as concentrations of MP detected (MPs/ul plasma).

### Blood chemistries/routine laboratory markers

Additional blood samples for routine analysis were obtained and analysed by the clinical chemistry Laboratory of the Karolinska University Hospital, Stockholm, Sweden.

### Statistical methods

Sample size in this descriptive, explorative study was based on a large effect size (f > 0.6) shown in previous similar studies, requiring at least 12 subjects in each of the three groups. The MP concentrations are presented as medians with upper and lower quartile values in parenthesis. None of the MP variables were normally distributed according to Shapiro-Wilks normality test. Between group differences were tested using the Kruskal-Wallis analysis and post hoc analyses by the Mann Whitney U test. Significance was accepted at *p* < 0.05. Regression analyses including MP concentrations, diabetes mellitus and eGFR were performed. Statistical analyses were performed using SPSS software, version 22 (SPSS Inc., Chicago, IL, USA).

## Results

Forty-seven patients were included in the study, and patient’s characteristics are summarized in Table 1. As can be seen, the group with normal renal function was younger and had less comorbidities, such as diabetes mellitus and hypertension, compared with the two groups with renal failure. The medical treatment was similar in the three groups. All patients were treated with antiplatelet therapy P2Y_12_ receptor antagonists (mainly clopidogrel; see Table I), and all but two patients were on ASA therapy. Ongoing statin treatment was the same in all groups.

**Fig. 1:**
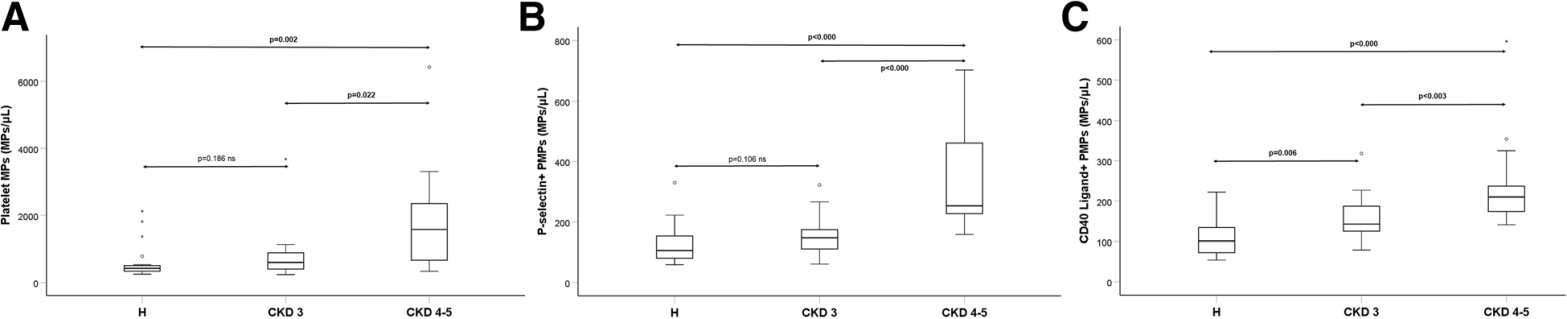
**a-c** Box plots of PMP concentrations for the three groups of patients with various degree of kidney function. Group H (*n* = 19); CKD 3 (*n* = 15); CKD 4–5 (*n* = 13)

**Fig. 2:**
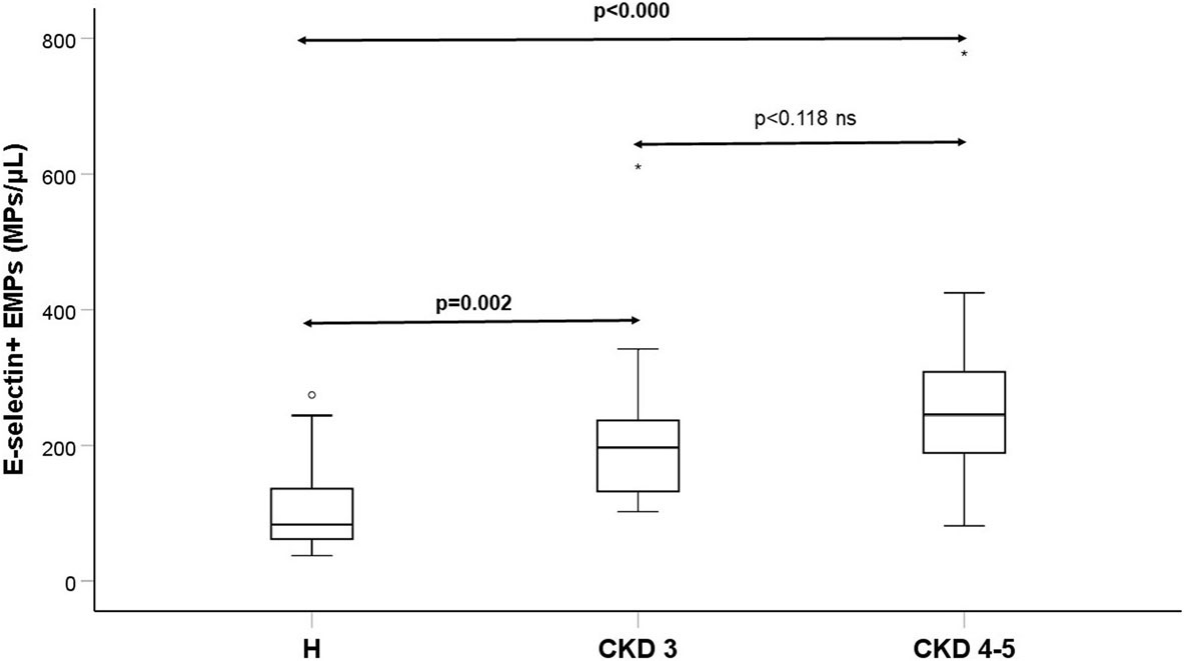
Box plot of EMP concentrations for the same three groups of patients as in Fig. 1

**Fig. 3:**
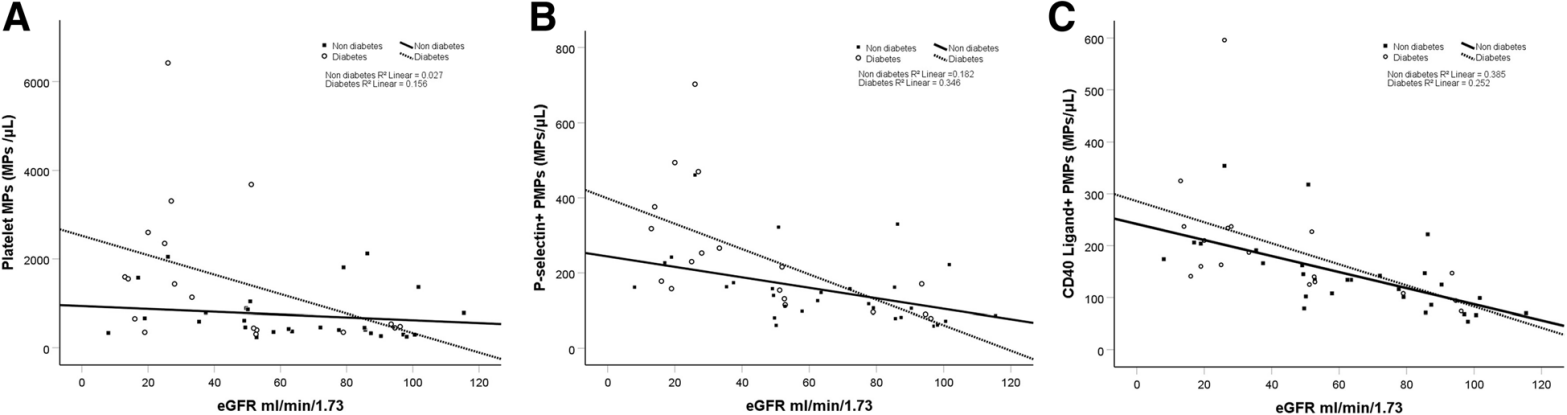
**a-c** Scatterplots of concentrations of all PMPs and eGFR. The regression lines shown are stratified for diabetes mellitus (DM) or not

**Fig. 4:**
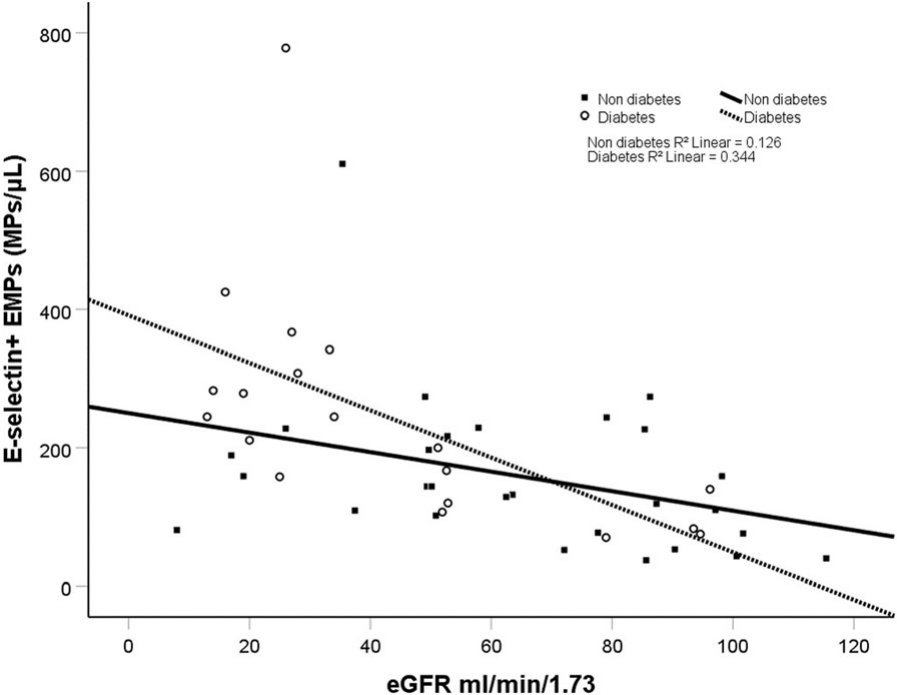
Scatterplot of concentration of E-selectin positive EMPs in relation to eGFR. The regression lines shown are stratified for diabetes mellitus (DM) or not

Table 2 and Fig. 1a-c and 2 show concentrations of MPs in the three groups. Figure 3a-c and 4 show scatterplots illustrating the correlation between eGFR and the MP concentrations in patients with and without diabetes mellitus diagnosis. The regression analyses showed correlations between eGFR and MP concentration, both in patients with and without diabetes mellitus.

**Table 2:** MP concentrations Median and interquartile range a = *p* value for comparison between the three groups. b = p value for comparison between the two groups listed

	H	CKD 3	CKD 4–5	*p*-value^a^
PMP (count/μL) (CD41)	424 (328–534)	600 (401–888)	1576 (666–2351)	0.001^**a**^ (CKD3 vs CKD 4–5, ***p*** **= 0.022**)^b^ (CKD3 vs H, *p* = 0.186) ^b^ (CKD4–5 vs H**,** ***p*** **= 0.002**) ^b^
P-selectin positive PMP (count/μL) (CD 62P)	106 (79–158)	147 (111–174)	253 (227–461)	< 0.001^**a**^ (CKD3 vs CKD 4–5, ***p*** **< 0.000**)^b^ (CKD3 vs H, *p* = 0.106) ^b^ (CKD4–5 vs H**,** ***p*** **< 0.000**) ^b^
CD40 ligand positive PMP (count/μL) (CD154)	101 (71–134)	142 (125–187)	210 (174–237)	< 0.001^**a**^ (CKD3 vs CKD 4–5, ***p*** **< 0.003**)^b^ (CKD3 vs H, ***p*** **= 0.006**) ^b^ (CKD4–5 vs H, ***p*** **< 0.000**)^b^
E-selectin positive MP (count/μL) (CD 62E)	83 (53–140)	197 (120–245)	245 (189–308)	< 0.001^**a**^ (CKD3 vs CKD 4–5, *p* < 0.118) ^b^ (CKD3 vs H, ***p*** **= 0.002**) ^b^ (CKD4–5 vs H, ***p*** **< 0.000**)^b^

Three-group comparisons using Kruskal Wallis method showed significant increases in CKD groups for the total concentrations of PMPs (CD41), for PMPs expressing platelet activation marker CD40 ligand (CD154) and P-selectin (CD62P) as well as EMPs expressing endothelial activation marker E-selectin (CD62E).

Post hoc analyses with two group comparisons were performed with Mann-Whitney U test, and showed that concentrations of PMPs expressing platelet activation marker CD40 ligand differed significantly between all three groups of patients with AMI. The concentration was highest in CKD 4–5; 210 (174–237; median and interquartile range), elevated in CKD 3; 142 (125–187) and lowest in the group with normal renal function, group H; 101 (71–134).

P-selectin positive PMPs were higher in the AMI group with CKD 4–5; 253 (227–461; *p* < 0.000), compared with H; 106 (79–158). We found a numerical trend towards higher P-selectin PMPs in CKD 3; 147 (111–174), compared with H; 106 (79–158), but this was not significant (*p* = 0.11).

Total concentrations of PMPs differed significantly between group H; 424 (328–534) and CKD 4–5; 1576 (666–2351; *p* = 0.002). Between group H and group CKD 3; 600 (401–888), the numerical differences observed when comparing the median concentrations of PMPs was not significant (*p* = 0.19).

Concentrations of CD62E positive (E-selectin) EMPs were significantly higher in both CKD groups: CKD 4–5; 245 (189–308) compared with H; 83 (53–140; *p* < 0.0001) and CKD 3; 197(120–245) compared with H (*p* < 0.002). Concentrations of EMPs appeared numerically higher in CKD 4–5 compared with CKD 3, but this was not significant (*p* = 0.118).

Routine laboratory markers showed that platelets and white blood cell counts were the same between the three groups of patients with AMI. Mean plasma albumin levels were 30 and 33 g/L in CKD 3 and CKD 4–5 respectively, and 35 g/L in H.

## Discussion

In this descriptive, explorative study, we measured circulating PMPs expressing platelet activation markers CD40 ligand (CD154) and P-selectin (CD62P), as well as levels of EMPs expressing E-selectin (CD62E), in patients who were hospitalized for an AMI and on dual antiplatelet treatment. The patients were divided in three groups according to renal function, and we found that the concentrations of PMPs expressing CD40ligand, and P-selectin, as well as EMPs expressing E-selectin, were significantly higher in patients with severe CKD. Our results are in agreement with the notion that MPs might be a novel pathway that contributes to the poor outcome after a cardiovascular event seen in CKD,

The possible link between MPs and inflammation, vascular dysfunction and pro-thrombosis, were demonstrated in an in vitro study, where PMP enriched blood were able to increase deposition of platelets and fibrin in human atherosclerotic vessels, PMPs hereby directly contributing to thrombosis formation [21].

In AMI, the thrombus formation at the site of injury is dependent on complex interactions between activated platelets, circulating PMPs, activated endothelial cells and the coagulation system [14]. In previous studies it has been shown that AMI patients have high levels of PMPs as well as EMPs [18, 31, 32]. In contrast, one study of ST-elevation AMI patients (STEMI) without CKD showed that PMP concentrations were not significantly different compared to healthy individuals, and the concentrations of PMPs were not associated to outcome [33]. However, in two other studies, high concentrations of both PMPs and EMPs were associated with the severity of AMI [34, 35] in non CKD patients. Another recent study in post-AMI patients found that elevated concentrations of P-selectin positive PMPs were associated with an increased risk for a new cardiovascular event [36]. There are currently no outcome studies published on PMPs in AMI patients with reduced renal function, but our results indicate that PMPs may be of particular interest in this patient group.

In our study the concentrations of PMPs expressing activation marker p-selectin as well as PMPs without any activation marker, were higher in the severe kidney disease group CKD 4–5, but not in the moderate kidney disease group CKD 3, compared with the group H without kidney disease. The reason for this might be simply a to small sample to discover true differences, or this finding might be partially explained by results found in a study of platelet reactivity. In this study of high post-treatment platelet reactivity (HPPR), patients with diabetes mellitus and CKD, treated with standard dual antiplatelet treatment, remaining high platelet reactivity was noticed only in severe CKD, not in moderate CDK. The authors speculate that there might exist a threshold after which renal dysfunction impact platelet reactivity [37].

Endothelial dysfunction is evident early in the atherosclerotic process and promotes chronic vascular inflammation commonly seen in CKD [38]. Loss of endothelium dependent vasodilatation is one important factor contributing to functional stiffening of the arteries and subsequent accelerated CVD and progression of CKD [39, 40]. Studies performed have shown a correlation between levels of EMPs and measures of endothelial function [26] and EMPs seem to be chronically high in CKD patients [15, 16, 23, 41]. However, there are as far as we know no study performed investigating the levels of EMPs post AMI comparing CKD to non CKD patients. Our results, with higher levels of EMPs in CKD patients post AMI, indicate a more advanced endothelial damage in these patients. Further studies with a prospective design are needed to evaluate EMPs role as a prognostic marker in this patient population.

Researchers from our group have previously shown that patients with non STEMI treated with aspirin or statin at admission did not differ in levels of PMPs compared to patients without treatment who were given a bolus of aspirin in the ambulance before admission [31]. However other studies have shown decreasing levels of PMPs after the initiation of antithrombotic treatment [32]. Initiation of statin treatment has also been shown to lower levels of PMPs expressing P-selectin [22, 42] as well as CD40 ligand PMPs [43]. It is therefore important to note that all patients in our study had received the same potent platelet inhibition with P2Y12 antagonists (clopidogrel, ticagrelor or prasugrel) and ASA, latest the day before the blood sampling, and that statin treatment was the same in the three groups. Levels of platelet count were also the same in the groups. Thus, the differences found in the present study were likely not due to different statin or antiplatelet treatment, but may indicate a reduced efficacy of P2Y12 receptor antagonists in CKD patients.

Age, smoking habits and gender differed between the three groups in this study, but the numbers where too few to perform a regression analysis. Previous work, however, have shown that none of these affect the concentrations of MPs in AMI patients without CKD [36]. In our study, hypertension was more common in CKD patients. Previous work has shown that concentrations of EMP and PMP are elevated in severe hypertension, but it is not known whether controlled hypertension, as in our patients, affect concentrations [44].

There was a difference in the proportion of prevalence of diabetes mellitus between the three groups with different eGFR. After adjusting for eGFR and diabetes mellitus, the between group MP differences remained.

Blood samples were taken 1–3 days after the admission for AMI, in the morning, fasting, which reduces diurnal variability. It has previously been shown that PMPs decrease 24 h after initiating ACS treatment, and further decrease after 6 months [31]. According to this there might be a gradual decrease of PMPs as day passes. At the same time, it is known that the delay in AMI patients before they seek acute care vary from hours to days, making it hard to determine the actual time of plaque rupture [45]. In our study, the severe kidney disease group CKD 4–5 had the highest PMPs, despite the fact that their samples were taken almost 1 day later compared with the other groups, supporting the notion that sample delay was not the cause of our findings. The variability 1–3 days, of which the samples were taken adds an uncertainty to our results, however as discussed above we argue that this variability does not explain our findings.

It is of importance to note that there was no difference in proportion of STEMI versus non STEMI in our three groups since previous papers reported that MP levels vary with type of AMI and infarction size [34, 35]. There were some differences in the proportion of patients undergoing percutaneous coronary intervention (PCI), with slightly fewer PCIs in the group with CKD 3. There are conflicting results regarding the effect on MP levels after PCI. Two studies [46, 47] that collected blood from the culprit lesion, show initially increased MPs, decreasing after PCI, interpreted as due to the initiation of antiplatelet treatment and the restored blood flow. Another study found decreased levels of PMPs, but augmented levels of EMPs, reflecting the acute endothelial injury after PCI [32]. Our results might be affected by these findings, since CKD group 3 had slightly less PCIs performed. Even so the differences were clearest between the control group and the group with CKD stage 4–5, which indicates that our results are not explained only due to PCI or not.

These findings all together indicates that a prospective outcome study with a larger sample size would be warranted.

### Limitations of the study

The main limitation of this explorative study is the small number of patients with a limited power to exclude smaller differences between the group, and limited power to adjust for potential confounders as discussed above. A second limitation is the lack of a control group without myocardial infarction.

## Conclusions

In patients with AMI, concentrations of PMPs expressing platelet activation markers CD40 ligand (CD154) and P-selectin (CD62P), as well as EMPs expressing markers of endothelial activation E-selectin (CD62E) were significantly higher in patients with severe CKD, compared with CKD 3 and normal kidney function patients, when adjusting for diabetes mellitus prevalence. This indicates further impaired endothelial function and higher platelet activation in CKD patients, despite concurrent dual antiplatelet and statin treatment. Our results highlight the need for further studies on antiplatelet treatment and efficacy in CKD patients, and the potential role of MPs as prognostic markers.

## Abbreviations

AMI: Acute myocardial infarction
CKD 3: Chronic kidney disease stage 3
CKD 4–5: Chronic kidney disease stage 4 and 5
CVD: Cardiovascular disease
eGFR: Estimated glomerular filtration
EMPs: Endothelial microparticles
MP: Microparticles
PMPs: Platelet microparticles
PPP: Platelet poor plasma; ACS: acute coronary syndrome
